# Causal Association Between Transferrin Saturation and Periodontitis: A Mendelian Randomization Study

**DOI:** 10.3290/j.ohpd.c_2421

**Published:** 2026-02-10

**Authors:** Jie Gao

**Affiliations:** a Jie Gao Lecturer, Department of Stomatology, Xi’an International Medical Center Hospital Affiliated to Northwest University, Xi’an 710061, Shaanxi, China.

**Keywords:** iron deficiency anemia, iron status, Mendelian randomization, periodontitis, transferrin saturation.

## Abstract

**Purpose:**

Iron deficiency anemia may influence the development of periodontitis bidirectionally. This study aimed to examine the causal association of iron deficiency anemia and iron status on the occurrence of periodontitis predicted through Mendelian randomization (MR) analysis.

**Materials and Methods:**

This two-sample MR study used summary data from large-scale genome-wide association studies of iron deficiency anemia (obtained through the meta-analysis of two large datasets), iron status, and periodontitis. Analysis was conducted using inverse variance weighted (IVW) as the main analysis and with weighted median, weighted mode, and MR-Egger regression methods as complementary analyses. Sensitivity analyses were evaluated using Cochran’s Q-test, MR-Egger regression, MR-PRESSO analysis, and leave-one-out analysis to assess the robustness and consistency of the results.

**Results:**

Genetic predictions indicated a statistically significant association between transferrin saturation as the exposure and periodontitis as the outcome (OR=1.23, 95%CI: 1.08-1.41, p=0.002). No causal associations were observed between the other exposures (iron deficiency anemia, serum iron, serum ferritin, and TIBC) (all p>0.05). Cochran’s Q-test showed no statistically significant heterogeneity, and the MR-Egger regression results suggested that this analysis was not influenced by horizontal pleiotropy. The MR-PRESSO results indicated that there were no outliers.

**Conclusion:**

The results suggest the presence of a positive causal association between transferrin saturation and periodontitis, but not iron deficiency anemia, serum iron, serum ferritin, and TIBC as exposures. Hence, the findings provide genetic evidence that anemia may be a potential cause for periodontitis, suggesting that attention to and management of patients’ systemic hematological status may be important in the prevention and comprehensive treatment of periodontal disease.

Periodontitis damages the underlying connective tissue attachment of the tooth through increasing periodontal pocket depth and alveolar bone loss.^44,45^ According to the Global Burden of Disease 2015 study, the worldwide prevalence of severe periodontitis was estimated at 7.4%,^31^ and the estimated prevalence of periodontitis of any severity is reported to be 50% among adults in the USA.^21^ Persons who are socially, economically, and racially marginalized experience a greater prevalence of periodontitis (and other oral diseases) due to factors including lack of access to and/or prohibitive cost of primary and preventative oral healthcare, lower education levels, and others.^21^ Untreated periodontitis can lead to tooth loss, impairing chewing function and esthetics, and complicated outcomes in various chronic conditions, including diabetes, cardiovascular diseases, and pregnancy,^32^ with a significant negative impact on a patient’s quality of life. Except for poor oral hygiene and smoking, certain systemic diseases and conditions, illicit drugs, genetic susceptibility, and some medications also might increase the risk of periodontitis. Genetics may also influence the development and progression of periodontitis.^37,46^ Today, periodontitis is considered a condition with systemic involvement through cytokines.^1,5,50^

One of these conditions could be iron deficiency anemia, which occurs when iron deficiency has progressed to iron-deficient erythropoiesis, which is the most common cause of anemia worldwide, accounting for about 50% of cases.^35^ Iron deficiency and iron deficiency anemia may occur due to an increased need for iron (e.g., during pregnancy), decreased iron intake (e.g., lack of iron sources in the diet), reduced iron absorption (e.g., coeliac disease), or loss of iron (e.g., bleeding).^12^ In some patients, iron deficiency anemia may be multifactorial or may coexist with other causes of anemia, especially anemia of inflammation.^19,30,39^ Studies suggested that the chronic inflammatory state caused by chronic periodontitis favors the development of anemia.^25^ On the other hand, anemia induces fatigue and immune depression, which could theoretically increase the susceptibility to the development of periodontitis. In addition, high transferrin saturation appears to be associated with severe periodontitis.^2^ Transferrin serves as the primary carrier for iron transport within the body, facilitating the delivery of iron to target cells via the circulatory system or the efflux of excess iron from cells.^20^ Elevated transferrin saturation increases iron bioavailability, promoting the proliferation of pathogenic bacteria, such as *Treponema*, and leading to dysbiosis, which in turn worsens periodontitis.^9^ Further research is essential to generate high-quality evidence concerning periodontitis, particularly focusing on its prevalence and implications within the general population.

Several large-scale genome-wide association studies (GWASs) performed in the last decades has generated a tremendous amount of data about the associations between single-nucleotide polymorphisms (SNPs) and phenotypes and diseases.^47,51^ The Mendelian randomization (MR) method uses the variations measured in genes to examine the causal association between an exposure and an outcome. MR mitigates the risks of reverse causation and confounding, thereby rectifying the potential misinterpretations inherent in the findings of observational epidemiological studies.^28^ Nevertheless, to be valid, MR must be performed under strict assumptions: 1) relevance (i.e., the genetic variant[s] being used as an instrument for exposure is associated with the exposure); 2) independence (i.e., there are no common causes of the genetic variant[s] and the outcome of interest); and 3) no horizontal pleiotropy (i.e., there is no independent pathway between the genetic variant[s] and the outcome other than through exposure).^28^ Two-sample MR uses the associations between SNPs and exposure and between SNPs and outcomes from different GWASs to combine them into causal associations.^18^ A special type of meta-analysis can be used to combine GWAS datasets to increase the power of the analysis,^54^ which was done here for iron deficiency anemia.

Given the available observational evidence, this study aimed to investigate the potential causal relationship between iron deficiency anemia and iron status-related factors as exposures and periodontitis as outcome using a two-sample MR approach. The null hypothesis was that genetically predicted anemia increases the risk of genetically predicted periodontitis.

## MATERIALS AND METHODS

### Study Design

**Fig 1:**
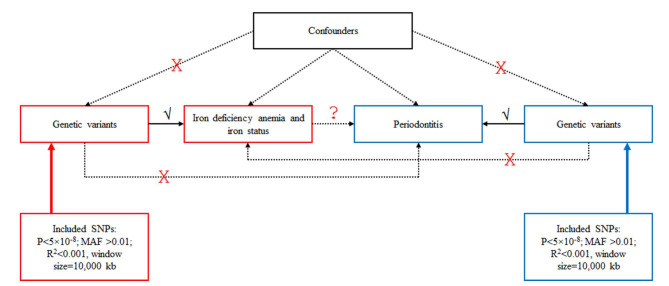
Schematic representation of the Mendelian randomization (MR) study. Genetic instrumental variables (IVs) (single-nucleotide polymorphisms [SNPs]) associated with the exposures (iron deficiency anemia and iron status-related traits) were identified from publicly available genome-wide association study (GWAS) datasets. These SNPs were then examined in the outcome dataset (periodontitis) to determine whether they were also associated with periodontitis. Since alleles are randomly distributed during gametogenesis and conception, MR can mimic a randomized trial to evaluate whether genetically predicted anemia causes genetically predicted periodontitis, without requiring detailed data from the same individuals on both anemia and periodontal disease.

This study used publicly available data from GWASs to investigate the causal association between iron deficiency anemia and iron status on periodontitis (Fig 1). All data used in this study are publicly available from studies that already adhered to the Declaration of Helsinki and Good Clinical Practices, so that no ethical approval was necessary. Based on the MR assumptions, this study assumed that the SNPs used as instrumental variables (IVs) for the exposure (iron deficiency anemia and iron status) are associated with the exposure, that there are no common causes to the SNPs and the outcome (periodontitis), and that there is no independent pathway between the SNPs and periodontitis other than through iron deficiency anemia and iron status.

### Data Source

The exposure and outcome datasets were from different, non-overlapping populations, fulfilling the requirements of a two-sample MR design. The GWAS data for periodontitis were from the GLIDE consortium GWAS database (https://data.bris.ac.uk/data/dataset/2j2rqgzedxlq02oqbb4vmycnc2). The data for iron deficiency anemia were meta-analyzed using the METAL software,^54^ combining GWAS data for iron deficiency anemia from the R10 version of the FinnGen project (https://www.finngen.fi/en) (GWAS ID finn-b-D3_ANAEMIA_IRONDEF; 3222 patients with iron deficiency anemia and 361,194 controls; all of European ancestry)^53^ and the UK Biobank (GWAS ID ukb-b-3355; 2066 patients with ICD10 D50.9 iron deficiency anemia, unspecified, and 460,944 controls; all of European ancestry). The merged data were then used for subsequent analysis. The GWAS data for iron status were from the Genetics of Iron Status Consortium 4 (https://www.decode.com/summarydata/) and included serum iron, serum ferritin, total iron-binding capacity (TIBC), and transferrin saturation (accessed on May 23, 2024). No individual-level clinical information is available in the GWAS datasets.

### Instrumental Variable Selection

The IVs included in this study had to adhere to the following criteria. Initially, SNPs statistically significantly associated with iron deficiency anemia and iron status in genome-wide analysis were screened with a threshold of p<5×10^-8^.^34^ SNPs with a minimum minor allele frequency (MAF) >0.01 were selected.^4^ SNPs showing linkage disequilibrium (LD) with an R^2^<0.001 and a window size of 10,000 kb were excluded. If the selected IVs were not present in the summary data for the outcome, proxy SNPs with a high LD (i.e., R^2^>0.8) were sought as substitutes.^48^ The F-value for each SNP in the IVs was calculated to assess the IV strength and to mitigate potential weak instrument bias between IVs and iron deficiency anemia/iron status. The F-value was calculated using F=R^2^×(N-2)/(1-R^2^). An F-value >10 indicated the absence of weak instrument bias.^52^

### Mendelian Randomization Analysis

The inverse variance weighted method (IVW) was the main analytical approach, and the potential causal association between exposure and outcome occurrence risk was quantified by computing the odds ratios (ORs) and 95% confidence intervals (CIs).^11^ The robustness of the associations identified by the IVW method was assessed using the MR-Egger,^6^ weighted median,^7^ and weighted mode^27^ methods. All analyses were performed using the “TwoSampleMR” package in R 4.0.5 (The R Project for Statistical Computing, www.r-project.org). Visualization was performed using forest and scatter plots.

### Sensitivity Analysis

The potential heterogeneity and horizontal pleiotropy were detected using sensitivity analyses. Heterogeneity among IVs was detected using Cochran’s Q test; p>0.05 indicated low heterogeneity and that the estimates among IVs were randomly fluctuating, with minimal impact on the IVW results.^8^ Considering that the third MR assumption calls for the absence of horizontal pleiotropy, the MR-Egger regression method was used to rule out horizontal pleiotropy.^8^ The MR pleiotropy residual sum and outlier (MR-PRESSO) analysis was used to detect potential outliers (SNPs with p<0.05), which were then removed to recalculate the causal association, hence correcting for horizontal pleiotropy.^8,49^ A leave-one-out analysis was used to assess the robustness and consistency of the results by sequentially removing one IV and recalculating.^10^

## RESULTS

### Inclusion of the Instrumental Variables

This MR analysis included five, 65, 30, 31, and 25 IVs for iron deficiency anemia, serum ferritin, serum iron, TIBC, and transferrin saturation as exposures, respectively. The IV selection and F-values are shown in Table 1. All F-values were >10, indicating no weak instrumental bias. Detailed information for all used IVs is shown in Supplementary Table A1.

**Table 1:** Instrumental variable selection

Exposure	IVs, n	Mean F-value	F-value range	Unmatching SNPs	Proxy SNPs, when available
Iron deficiency anemia	5	95.13	30.84-228.34	1	No replacement
Serum iron	30	100.81	29.72-1262.99	1	No replacement
Serum ferritin	65	78.43	29.73-519.49	8	rs10801913-> rs10754329
					rs1260326-> rs780094
					rs6760824-> rs4666145
					rs590097-> rs604126
					rs71537957-> rs35435439
					rs17676097-> rs17676067
					rs45520632-> rs117753546
TIBC	31	87.66	30.51-698.55	4	rs28986298-> rs362521
					rs2236252-> rs12480826
Transferrin saturation	25	167.14	29.73-1408.47	3	rs7648210-> rs9822847
					rs1233602-> rs381808
					rs3130059-> rs909253
IV: instrumental variables; SNP: single-nucleotide polymorphism; TIBC: total iron-binding capacity.

### Mendelian Randomization Results

**Fig 2:**
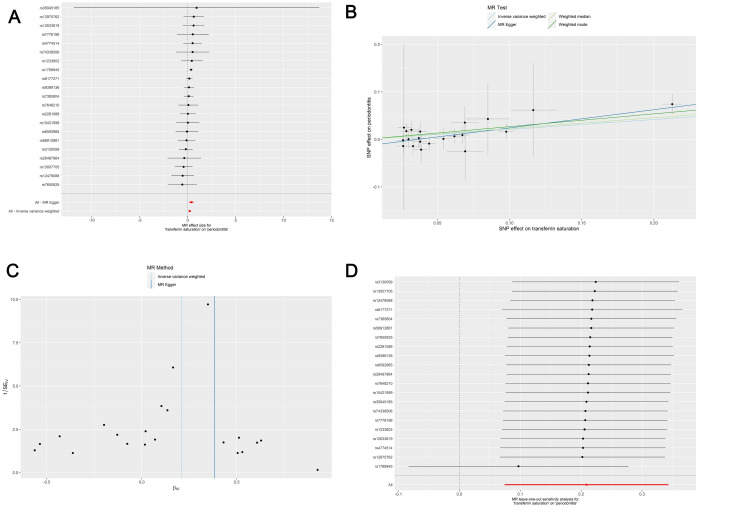

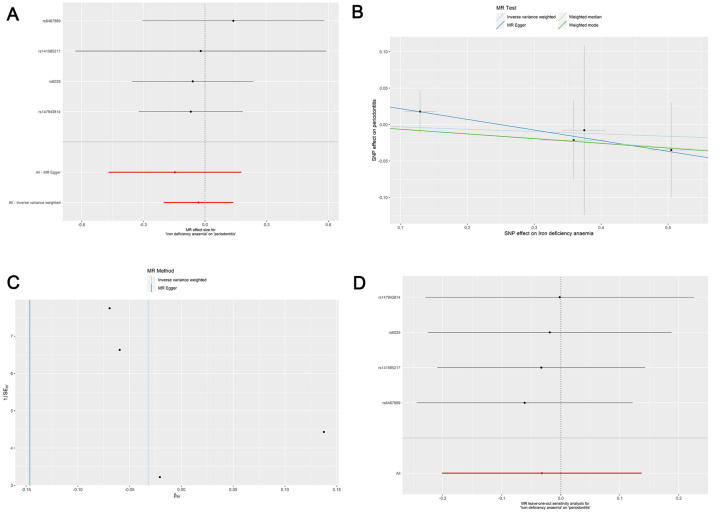

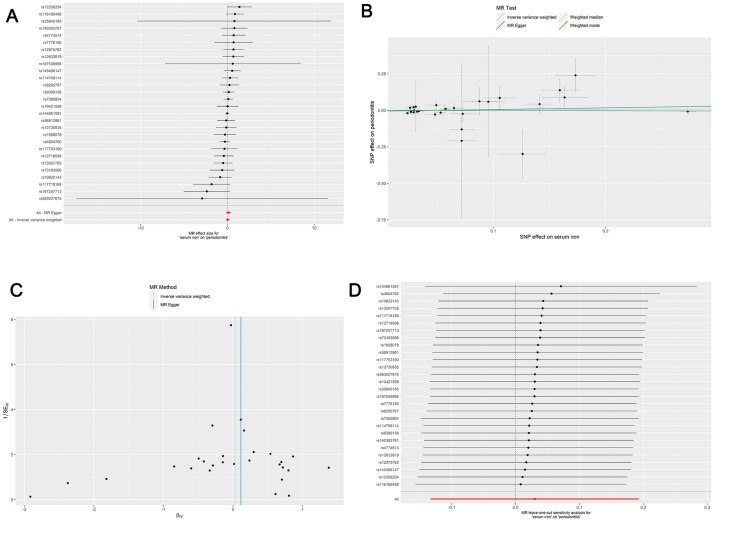

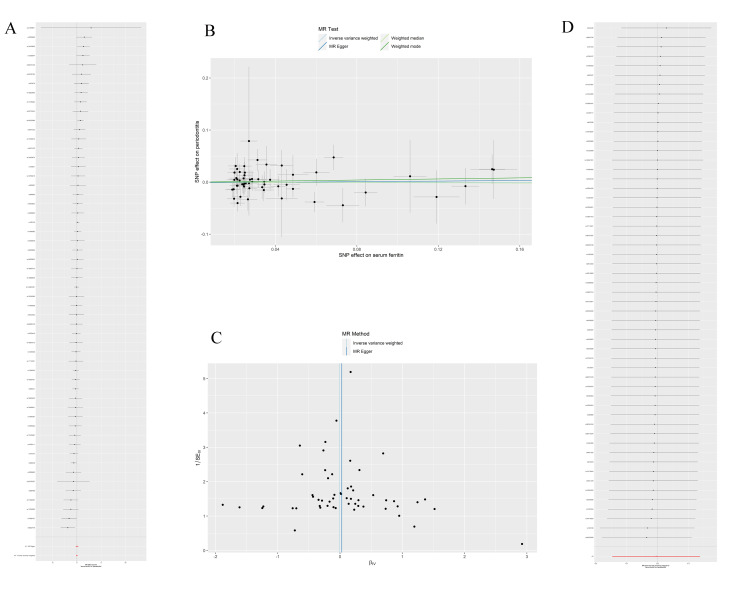

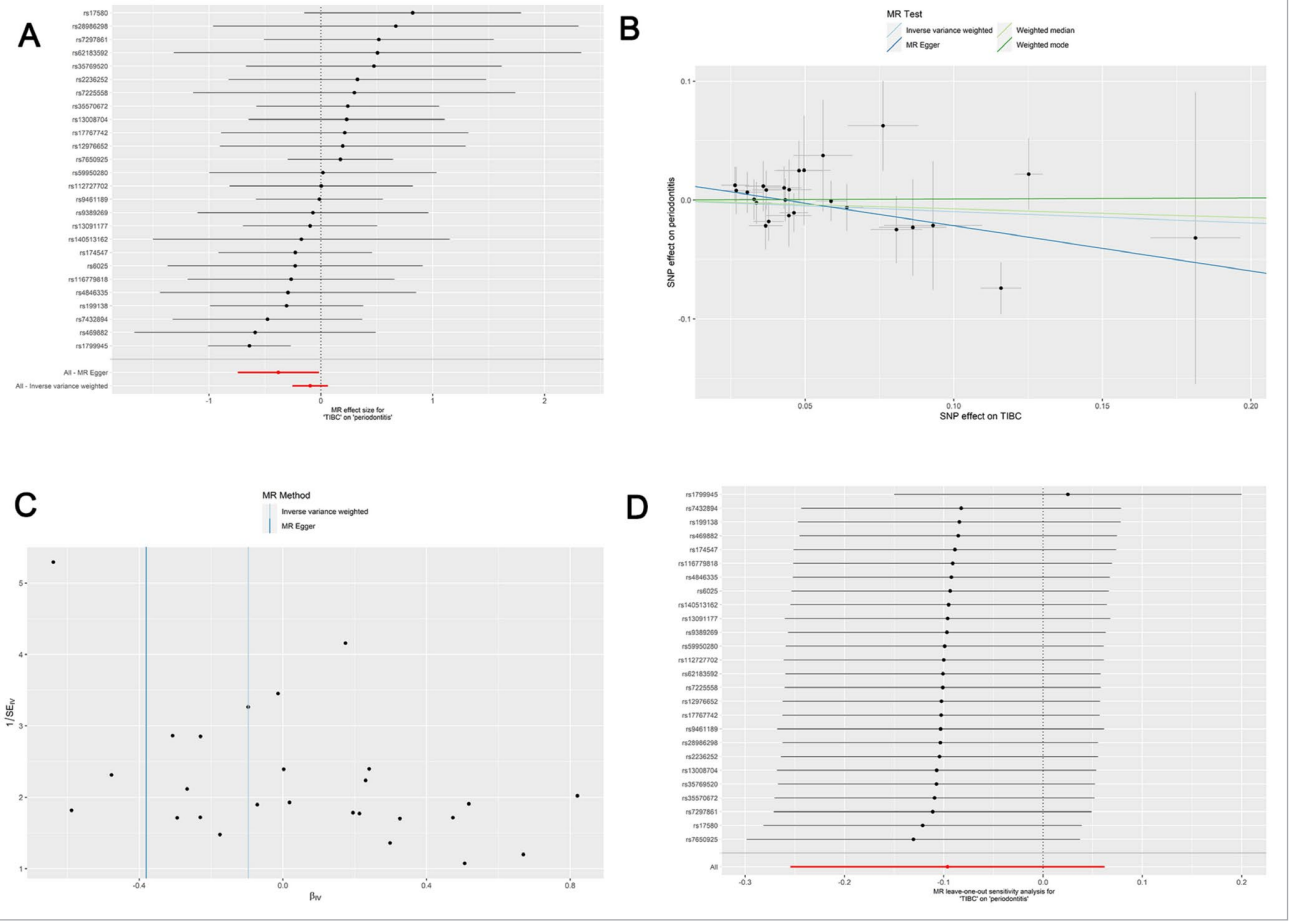
Forest plot (A), scatter plot (B), funnel plot (C), and left-one-out forest plot (D) of the Mendelian randomization analysis for the causal association of genetically predicted transferrin saturation on genetically predicted periodontitis.

The primary inverse variance weighted (IVW) method was applied first to evaluate the overall causal effect of anemia on periodontitis. The genetic prediction results showed a statistically significant association between transferrin saturation as exposure and periodontitis as outcome (OR=1.23, 95%CI: 1.08-1.41, p=0.0021) (Table 2 and Fig 2). The results of the three other MR methods were consistent with the IVW results. No causal associations were observed between the other exposures (iron deficiency anemia, serum iron, serum ferritin, or TIBC) and the outcome (all p>0.05) (Table 2 and Supplementary Figs A1 to A4).

**Table 2:** The causal association between genetically predicted iron deficiency anemia and the risk of periodontitis

Outcome	Exposure	n SNP	Methods	OR (95%CI)	p
Periodontitis	Iron deficiency anemia	4	IVW	0.97 (0.82–1.15)	0.710
Periodontitis	Iron deficiency anemia	4	MR Egger	0.86 (0.63–1.19)	0.468
Periodontitis	Iron deficiency anemia	4	Weighted median	0.94 (0.77–1.14)	0.530
Periodontitis	Iron deficiency anemia	4	Weighted mode	0.94 (0.74–1.18)	0.622
Periodontitis	Transferrin saturation	21	IVW	1.23 (1.08–1.41)	0.002
Periodontitis	Transferrin saturation	21	MR Egger	1.47 (1.17–1.83)	0.003
Periodontitis	Transferrin saturation	21	Weighted median	1.26 (1.05–1.5)	0.013
Periodontitis	Transferrin saturation	21	Weighted mode	1.31 (1.11–1.54)	0.005
Periodontitis	Serum ferritin	55	IVW	1 (0.86–1.15)	0.951
Periodontitis	Serum ferritin	55	MR Egger	1.03 (0.79–1.34)	0.836
Periodontitis	Serum ferritin	55	Weighted median	0.99 (0.79–1.24)	0.927
Periodontitis	Serum ferritin	55	Weighted mode	1.05 (0.79–1.39)	0.723
Periodontitis	Serum iron	28	IVW	1.03 (0.88–1.21)	0.720
Periodontitis	Serum iron	28	MR Egger	1.12 (0.87–1.44)	0.382
Periodontitis	Serum iron	28	Weighted median	0.99 (0.79–1.23)	0.895
Periodontitis	Serum iron	28	Weighted mode	0.98 (0.8–1.21)	0.875
Periodontitis	Tibc	26	IVW	0.91 (0.78–1.06)	0.233
Periodontitis	Tibc	26	MR Egger	0.68 (0.48–0.98)	0.051
Periodontitis	Tibc	26	Weighted median	0.93 (0.73–1.18)	0.541
Periodontitis	Tibc	26	Weighted mode	1.01 (0.6–1.71)	0.974
OR: odds ratio; CI: confidence interval; IVW: inverse variance weighted; TIBC: total iron–binding capacity.

A series of sensitivity analyses was conducted to ensure that our results were robust and not influenced by outlier variants or horizontal pleiotropy (i.e., genetic variants affecting multiple traits), including Cochran’s Q-test (heterogeneity), MR-Egger regression (horizontal pleiotropy), and MR-PRESSO (outliers and horizontal pleiotropy). Cochran’s Q-test showed no statistically significant heterogeneity, and the MR-Egger regression results suggested that this analysis was not influenced by horizontal pleiotropy (Table 3). The MR-PRESSO results indicated that there were no outliers (Table 4). The leave-one-out analysis suggested that no single SNP drove the results (Fig 2 and Supplementary Figs A1–S4).

**Table 3:** Results of heterogeneity and pleiotropy testing for the instrumental variables of different exposures for periodontitis as the outcome

Outcome	Exposure	Heterogeneity	Pleiotropy
Q statistic (IVW)	p	MR-Egger Intercept	p
Periodontitis	Transferrin saturation	11.119	0.943	-0.014	0.071
Periodontitis	Serum ferritin	50.946	0.593	-0.001	0.776
Periodontitis	Serum iron	24.78	0.587	-0.006	0.396
Periodontitis	Iron deficiency anemia	0.683	0.877	0.036	0.501
Periodontitis	TIBC	21.692	0.653	0.016	0.101
IVW: inverse variance weighted; TIBC: total iron-binding capacity.

**Table 4:** Results of the MR-PRESSO test of different exposures for periodontitis as the outcome

Outcome	Exposure	Raw	Outlier corrected	Global p	Outliers	Distortion p
OR (95%CI)	p	OR (95%CI)	p
Periodontitis	Iron deficiency anemia	0.9684 (0.8932–1.0499)	0.4927	NA (NA–NA)	NA	0.89	NA	NA
Periodontitis	Serum ferritin	0.989 (0.8627–1.1338)	0.8748	NA (NA–NA)	NA	0.592	NA	NA
Periodontitis	Serum iron	1.0256 (0.8801–1.195)	0.7488	NA (NA–NA)	NA	0.671	NA	NA
Periodontitis	TIBC	0.9048 (0.7837–1.0446)	0.1839	NA (NA–NA)	NA	0.558	NA	NA
Periodontitis	Transferrin saturation	1.2064 (1.089–1.3365)	0.0016	NA (NA–NA)	NA	0.806	NA	NA
OR: odds ratio; CI: confidence interval; TIBC: total iron–binding capacity.

## DISCUSSION

The present MR study aimed to examine the causal association of iron deficiency anemia and iron status with the occurrence of periodontitis. The results suggest the presence of a positive causal association between transferrin saturation and periodontitis, but not iron deficiency anemia, serum iron, serum ferritin, or TIBC as exposures. The results of the sensitivity analysis further proved the robustness of the current findings.

Periodontitis as a cause of anemia (i.e., the so-called anemia of inflammation) is well-documented and arises from the chronic inflammatory state that increases red blood cell turnover and decreases hematopoiesis.^25^ As early as the 1960s, it was postulated that chronic fatigue and impaired immunity induced by chronic anemia would predispose individuals to various chronic infections, including periodontitis caused by a decreased immunity to the bacteria colonizing the gums.^33^ Chakraborty et al^13^ later showed that patients with iron deficiency anemia and periodontitis had more severe gingival breakdowns than patients with periodontitis without anemia and that a reduced serum superoxide dismutase activity (i.e., increased systemic oxidative stress) was involved in the process. Indeed, such impaired immune functions can facilitate gingival invasion by opportunistic pathogens like *Porphyromonas gingivalis*, which is a key pathogen in periodontal disease.^29^ Even a slight increase in *P. gingivalis* within the oral microbiome can disrupt the host immune responses and produce abundant inflammatory exudates as nutrients for other commensal bacteria, significantly promoting the onset and progression of periodontal disease, leading to alveolar bone resorption and tooth loss.^29,41^ Several destructive mechanisms of *P. gingivalis* are involved in periodontitis. For instance, *P. gingivalis* facilitates biofilm maturation, which protects bactera and makes their clearance more difficult, reducing the efficacy of antimicrobial agents.^3^ In addition, the inflammatory factors produced during *P. gingivalis* proliferation trigger destructive inflammatory cascades in periodontal tissues, causing damage to periodontal supportive structures and tooth loss.^29,41^ *P. gingivalis* also stimulates the secretion of matrix metalloproteinases (MMPs) by immune and mucosal cells, further degrading periodontal tissues.^22,29,41^

Nevertheless, in the present MR study, genetically predicted iron deficiency anemia had no causal association with periodontitis (even using the combined data from two large GWASs through meta-analysis), nor did iron status indices, including serum iron, serum ferritin, and TIBC. On the other hand, genetically predicted transferrin saturation had a causal association with genetically predicted periodontitis, observed using IVW and confirmed using the complementary MR analyses. Transferrin is a plasma glycoprotein responsible for ferric-ion delivery and is the most critical ferric pool in the body, since all plasma iron is bound to transferrin. Transferrin is the link between reticuloendothelial iron release and bone marrow uptake to meet the needs of hematopoiesis.^24,38^ Increased transferrin saturation has been associated with severe periodontitis, as increased non-transferrin-bound iron bioavailability promotes pathogenic bacteria growth.^9^ The transferrin receptor 2 also plays a role in periodontitis-induced alveolar bone loss.^36^ Increased transferrin saturation is linked to periodontitis risk. Monitoring transferrin saturation levels may help identify individuals at higher risk of developing periodontitis, potentially guiding early intervention strategies. Further research is needed to elucidate the underlying mechanisms.

Of note, a study was recently published using the same datasets for iron status and periodontitis as the present study, yet reaching different conclusions.^56^ That paper examined a wide range of dental diseases, while the present study focused only on periodontitis. Notwithstanding, the present study performed a multi-dataset meta-analysis for iron deficiency anemia, which was not performed in aforementioned study.^56^ The present study observed that transferrin saturation was causally associated with periodontitis, while the previous study reported that TIBC was a protective factor for periodontitis. Despite using the same datasets, the differences in iron status results may stem from the differences in the selection methods for IVs and results of the sensitivity analyses, leading to different SNPs being used for the MR analyses. However, the results are generally consistent, and the statistically significant results are not completely contradictory since they concern different biomarkers. It is possible that differences in IV selection affected statistical power; specifically, our study may have lacked the power to observe the significant results for TIBC reported by the previous study,^56^ while the previous study might not have reached the power necessary to observe the significant association with transferrin saturation found in this study. Additional studies would be necessary for confirmation. Nevertheless, multiple biochemical and population studies support a mechanistic and observational link between transferrin saturation and periodontal pathology. A clinical series in patients with sickle-cell anemia found that serum transferrin saturation > 45% was associated with 1.9-fold higher odds of rapid periodontitis progression. Likewise, an epidemiologic analysis in postmenopausal women reported that high ferritin and transferrin saturation levels were statistically significantly correlated with greater periodontitis severity and larger proportions of sites with clinical attachment loss > 4  mm. Mechanistic work further demonstrates that subgingival pathogens such as *Porphyromonas gingivalis* and *Treponema denticola * can acquire iron from transferrin degradation, promoting biofilm growth and tissue destruction in individuals with elevated transferrin saturation. These observations support the present study in that increased non-transferrin-bound iron bioavailability promotes pathogenic bacteria growth.^9,16,17^ Transferrin acts as the main ferric-ion transporter, regulating systemic and local iron distribution between macrophages of the reticuloendothelial system and hematopoietic tissue. Experimental work shows that transferrin receptor 2 participates in alveolar bone responses to inflammation, consistent with pathways linking iron signaling and osteoclast activity.^9^

Higher transferrin saturation indicates elevated circulating iron bound to transferrin, reflecting a state of iron overload or inefficient sequestration. Excess transferrin-bound iron enhances the generation of reactive oxygen species (ROS) through Fenton chemistry, leading to oxidative stress and damage of gingival epithelial and connective tissues. Iron overload also promotes activation of inflammatory pathways, particularly via IL‑6-hepcidin axis dysregulation, linking transferrin saturation increases to systemic and local inflammatory burden. This environment fosters periodontal pathogen proliferation, as *Porphyromonas gingivalis* and *Prevotella* species can exploit transferrin as an iron source, amplifying tissue destruction and inflammatory response.^15,26,42,43,55^ The absence of causal links with ferritin, serum iron, TIBC, or iron-deficiency anemia in the MR analysis indicates that the mechanistic risk is not driven by whole-body iron stores per se, but by the fraction of iron that is bioavailable and redox-active (transferrin saturation), capable of fueling oxidative stress and microbial growth in periodontal microenvironments.^23,55,56^ Clinically, this result implies that monitoring transferrin saturation might provide insight into systemic metabolic states that predispose patients to periodontitis, particularly in those without overt iron overload diseases. It also suggests that therapeutic modulation of iron handling, such as controlling dietary iron intake or oxidative stress, might have adjunctive value in periodontal disease prevention and management. These findings highlight a novel potential biomarker (transferrin saturation) and pathophysiological link between systemic iron metabolism and oral inflammatory disease.^15,26,42,43,55^ From a clinical perspective, our findings carry important implications. They suggest that when evaluating the systemic condition of patients with periodontitis, clinicians may need to look beyond the conventional diagnosis of “anemia” and pay closer attention to indicators of iron metabolism imbalance, particularly transferrin saturation. For patients with refractory or severe periodontitis, assessing iron metabolic status may help identify potential systemic risk factors. Future prospective clinical studies are warranted to determine whether modulating iron metabolism could serve as a novel adjunctive strategy to improve periodontal treatment outcomes. Moreover, these findings highlight the importance of interdisciplinary collaboration between dental and medical professionals in managing oral diseases related to systemic metabolic conditions.

Our study had several strengths. The first was the use of GWAS data from thousands of patients to evaluate the genetically predicted causal association between exposures and an outcome. Furthermore, a meta-analysis was used to combine two large datasets,^54^ one from Finland and one from the UK, to increase the power of the analysis. Besides, the stringent criteria for the selection of IVs in our MR study ensured adherence to the three fundamental principles.^40^ Additionally, the employment of multiple sensitivity analyses fortifies the robustness of our findings. However, the study also had limitations. The GWAS data in the present study were from European populations of European ancestry. Given the genetic variability among continents, the generalizability of the results obtained here to other populations is unknown. Additionally, the static nature of genetic information in MR studies might overlook dynamic changes in protein expression influenced by age, gender, body weight, disease progression, or other physiological factors, which could not be completely excluded.

## CONCLUSION

This MR analysis suggests the presence of a causal association between transferrin saturation and periodontitis. In light of these positive results, transferrin saturation warrants further investigation in the pathogenesis of periodontitis.

## Appendix

Appendix Table A1 Detailed information of instrumental variables used in MR analyses (https://www.quintessence-publishing.com/quintessenz/journals/articles/downloads/ohpd_2025_8116_gao_appendix.xlsx)

**Fig A1:** Forest plot (A), scatter plot (B), funnel plot (C), and left-one-out forest plot (D) of the Mendelian randomization analysis for the causal association of genetically predicted iron deficiency anemia on genetically predicted periodontitis.

**Fig A2:** Forest plot (A), scatter plot (B), funnel plot (C), and left-one-out forest plot (D) of the Mendelian randomization analysis for the causal association of genetically predicted serum iron on genetically predicted periodontitis.

**Fig A3:** Forest plot (A), scatter plot (B), funnel plot (C), and left-one-out forest plot (D) of the Mendelian randomization analysis for the causal association of genetically predicted serum ferritin on genetically predicted periodontitis.

**Fig A4:** Forest plot (A), scatter plot (B), funnel plot (C), and left-one-out forest plot (D) of the Mendelian randomization analysis for the causal association of genetically predicted total iron-binding capacity on genetically predicted periodontitis.
